# Sudden cardiac death: focus on the genetics of channelopathies and cardiomyopathies

**DOI:** 10.1186/s12929-017-0364-6

**Published:** 2017-08-15

**Authors:** Simona Magi, Vincenzo Lariccia, Marta Maiolino, Salvatore Amoroso, Santo Gratteri

**Affiliations:** 1 Department of Biomedical Sciences and Public Health, School of Medicine, University “Politecnica delle Marche”, Via Tronto 10/A, 60126 Ancona, Italy; 2 Department of Health Sciences, University “Magna Graecia”, 88100 Catanzaro, Italy

**Keywords:** Cardiomyopathies, Channelopathies, Caveolins, Genetics, Sudden cardiac death

## Abstract

Sudden cardiac death (SCD) describes a natural and unexpected death from cardiac causes occurring within a short period of time (generally within 1 h of symptom onset) in the absence of any other potentially lethal condition. Most SCD-related diseases have a genetic basis; in particular congenital cardiac channelopathies and cardiomyopathies have been described as leading causes of SCD. Congenital cardiac channelopathies are primary electric disorders caused by mutations affecting genes encoding cardiac ion channels or associated proteins, whereas cardiomyopathies are related to mutations in genes encoding several categories of proteins, including those of sarcomeres, desmosomes, the cytoskeleton, and the nuclear envelope. The purpose of this review is to provide a general overview of the main genetic variants that have been linked to the major congenital cardiac channelopathies and cardiomyopathies. Functional alterations of the related proteins are also described.

## Background

Sudden death (SD) is defined by clinicians as a natural and unexpected death that occurs within a short period of time (generally less than 1 h from the onset of acute symptoms) in an apparently healthy person or in a subject whose disease is not sufficiently severe to predict a fatal outcome. The most challenging point is that post-mortem examination often fails to demonstrate an adequate cause of death [1]. Indeed, approximately 85% of all SD are of cardiac origin, otherwise known as sudden cardiac death (SCD). Although SCD is a leading cause of death in western countries, many deaths remain largely unexplained [1–3]. SD involving infants under 1 year of age is referred as sudden infant death syndrome (SIDS). In general, SIDS occurs during sleep and remains unexplained after a thorough investigation that includes full autopsy, examination of the death scene, and review of the clinical history [4]. Many pathophysiologic mechanisms have been proposed for SIDS, including respiratory dysfunction, cardiorespiratory instability, and inborn errors of metabolism, however definitive pathogenic mechanisms precipitating an infant’s sudden death are still uncertain [5]. Arrhythmias related to cardiac channel mutations have been proposed as the pathogenic basis for an estimated 5 to 10% of SIDS cases involving Caucasian infants [5]. Such mutations mainly affect the *SCN5A* gene and result in phenotypic changes in this sodium channel [6]. In addition, the ethnic-specific common cardiac sodium channel polymorphism S1103Y-SCN5A has been associated with African American SIDS and sudden cardiac death in young black adults [5, 7, 8], reinforcing the hypothesis that genetic issues may be strikingly involved in SIDS.

Although the actual incidence of SCD is highly variable, recent prospective studies from multiple sources in the United States, Europe, and China have estimated that SCD rates in the general population range from 50 to 100 per 100,000 people annually [9]. The annual incidence of SCD increases as a function of advancing age; specifically, it is 100-fold lower in individuals < 30 years old (0.001%) than in those > 35 years of age [10–14]. However, the incidence is higher in men than in women at any age [9]. SCD has been shown to occur most frequently between birth and 6 months of age (SIDS) and again between 45 and 75 years of age [3].

The aetiologies of SCD are very diverse. However, certain diseases are known to play significant roles in its pathogenesis. In particular, congenital cardiac channelopathies and inherited cardiomyopathies have been described as leading causes of SCD.

Congenital cardiac channelopathies are caused by mutations affecting genes encoding membrane ion channels (sodium, potassium or calcium channels) or cellular structures that affect Ca^2+^ availability [15, 16]. Major cardiac channelopathies include long-QT syndrome (LQTS), short-QT syndrome (SQTS), Brugada syndrome (BrS), and catecholaminergic polymorphic ventricular tachycardia (CPVT) [15–17].

Cardiomyopathies are primarily related to cardiac structural abnormalities [18] that lead to arrhythmias. Although the causes of cardiomyopathy are varied, it is widely accepted that the disease may depend on genetic alterations in structural proteins, including those of sarcomeres, desmosomes, and the cytoskeleton [17–19]. The most common SCD-related cardiomyopathies in young children and adults include hypertrophic cardiomyopathy (HCM), dilated cardiomyopathy (DCM), restrictive cardiomyopathy (RCM), arrhythmogenic right ventricular cardiomyopathy (ARVC), and left ventricular noncompaction (LVNC) [18, 20].

Recent investigations of SCD have focused on *CAV3* genetic variants. The *CAV3* gene encodes the protein caveolin-3 [21, 22], a member of the caveolin family, which in humans also includes caveolin-1 and caveolin-2. Caveolins are the main constituents of caveolae, small plasma invaginations composed of cholesterol and other lipids that contain several protein complexes involved in signalling and vesicular trafficking [21]. Caveolin-3 is selectively expressed in cardiac caveolae, where it represents the main scaffolding protein [23]. Recent studies have identified *CAV3* mutations in subjects affected by LQTS, SIDS, and HCM [5, 24, 25].

Herein, we summarize the current genetic understanding of the main congenital diseases associated with SCD, with a specific focus on *CAV3* genetic variants.

## Congenital cardiac channelopathies

At the cardiac level, the perfect interplay between sodium, calcium, and potassium ions results in a heartbeat. Specific ion channels allow these ions to cross the myocardial membrane. Mutations in genes encoding these specific channels or associated proteins may impair ionic conduction, leading to channelopathies and life-threatening arrhythmias. As mentioned above, the main cardiac SCD-linked channelopathies are LQTS, SQTS, BrS, and CPVT.

### Long-QT syndrome

Long-QT syndrome (LQTS) is an inherited arrhythmia characterized by electrocardiographic prolongation of the QT interval. Clinical diagnosis relies on both the electrocardiographic presence of a prolonged QTc (QTc ≥ 480 ms in repeated 12-lead ECG, although a QTc ≥ 460 ms is sufficient in the presence of unexplained syncope [26]) and a comprehensive evaluation based on personal history, family history, and multiple electrocardiographic findings [27, 28]. LQTS patients are at risk of a polymorphic ventricular tachycardia called torsade de pointes, which can induce episodes of syncope and culminate in SD [15]. QT prolongation and torsade de pointes susceptibility are often related to ion channel dysfunctions that affect cellular repolarization. As shown in Table 1, prolonged cellular repolarization may be due to a decrease in outward potassium currents or an enhancement in the inward sodium current I_Na_. At present, 16 types of LQTS have been described (Table 1). The most common forms of LQTS are LQTS1, LQTS2, and LQTS3. Indeed, comprehensive open reading frame analysis of these three canonical LQTS-causative genes—*KCNQ1*-encoded Kv7.1 channel subunit (LQT1), *KCNH2*-encoded Kv11.1 (LQT2), and *SCN5A*-encoded Nav1.5 (LQT3)—yields putative LQTS-associated mutations in 75% of clinically definite LQTS cases [29]. Specifically, LQTS1, the most common mutation, accounts for approximately 35% of genotype-positive LQTS. LQTS1 results from a loss-of-function mutation of *KCNQ1*, the gene encoding the α- subunit of the voltage-gated potassium channel that mediates the slow component of the delayed rectifier potassium current (I_Ks_). Loss-of-function mutation of *KCNH2* affects the rapidly activating component of the delayed rectifying potassium current (I_Kr_) and is related to the second-most common form of LQTS (LQTS2, found in 30% of patients with LQTS). LQTS3 is the third-most common form (found in 10% of patients with LQTS). It results from a heterozygous gain-of-function mutation of the *SCN5A* gene, which encodes the α-subunit of the cardiac sodium channel Na_v_1.5. LQT3 has also been linked to both SIDS and autopsy-negative sudden unexplained death in childhood [5, 6].

**Table 1:** Pathogenic gene mutations associated with LQTS variants

Mutated gene	Encoded protein	Functional alteration	LQTS variant
*KCNQ1*	α-subunit of the voltage-gated potassium channel that mediates the slow component of the delayed rectifier potassium current (I_Ks_)	Reduction of I_Ks_, with subsequent prolonged repolarization of the action potential [111–113]	LQTS1
*KCNH2*	α-subunit of the voltage-gated potassium channel that mediates the rapidly activating component of the delayed rectifying potassium current (I_Kr_)	Reduction of I_Kr_ and delay in cardiac repolarization which leads to a prolonged QT interval [114]	LQTS2
*SCN5A*	α-subunit of the cardiac sodium channel Na_v_1.5	Gain-of-function. *SCN5A* variants induce an increased late inward Na_v_1.5 current, which slows cardiac repolarization and causes prolongation of the QT interval [115]	LQTS3
*ANK2*	Ankyrin-B, a protein involved in the coordinated assembly of the Na^+^/K^+^ ATPase, the Na^+^/Ca^2+^ exchanger, and the inositol triphosphate receptor	Calcium homeostasis impairment that prolongs repolarization [116]	LQTS4
*KCNE1*	β-subunit of Mink	Impairment of multimeric channel complex stability [117, 118]	LQTS5
*KCNE2*	β-subunit of MiRP1	Impairment of multimeric channel complex stability [119]	LQTS6
*KCNJ2*	Inward rectifier potassium channel Kir2.1 (I_K1_)	Impaired potassium current [120]	LQTS7
*CACNA1C*	L-type calcium channel	Impaired open-state voltage-dependent inactivation of the L-type calcium channel [121]	LQTS8
*CAV3*	Caveolin-3, the main scaffolding protein of cardiac caveolae	Gain-of-function increase in late sodium current [25]	LQTS9
*SCN4B*	β-subunit of the sodium channel	Gain-of-function increase in late sodium current [122]	LQTS10
*AKAP9*	Kinase-A anchor protein-9	Reduced interaction with KCNQ1 [123]	LQTS11
*SNTA1*	α1-syntrophin protein	Gain-of-function increase in late sodium current [124]	LQTS12
*KCNJ5*	Cardiac G-protein-coupled inward rectifier potassium channel subtype 4	Ventricular repolarization abnormality resulting in the prolongation of corrected QT and QT-peak intervals [125]	LQTS13
*CALM1*	Calmodulin, a protein involved in calcium-dependent inactivation of the L-type calcium channel and ryanodine channel stabilization, thus affecting overall intracellular calcium levels	Disruption of calcium-ion binding to the protein [126]	LQTS14
*CALM2*	Calmodulin	Disruption of calcium-ion binding to the protein [127]	LQTS15
*CALM3*	Calmodulin	Disruption of calcium-ion binding to the protein [128]	LQTS16
*TRDN*	Triadin	Impairment of cardiac calcium release that affects excitation-contraction coupling and leads to cardiac arrhythmias [34]	LQTS16

The additional minor LQTS genes comprise less than 5% of LQTS cases [29]. Depending on the affected ion channel, ventricular myocardial action potential changes along with the distribution of channels among the myocardium layers. Interestingly, it has been shown that QT interval duration does not vary among the different genotypes, whereas multiple ST-segment and T-wave morphologies have been associated with a particular genotype [30]. Such genotype-phenotype correlation has been described for the three most common LQTS genotypes [31]. In particular, patients with LQTS1 classically have a broad-based T-wave and usually have syncope or SD during physical exercise. LQTS2 patients tend to have notched or low-amplitude T-waves. They classically have syncope or SD with sudden auditory stimuli or strong emotions. Due to delayed opening of the sodium channel, LQTS3 patients have late-peaked T-waves, long, flat ST segments, a tendency towards bradycardia, and a higher incidence of SD during sleep [31].

Patients carrying two abnormal LQTS genes usually demonstrate a more severe clinical phenotype and are at higher risk of SD [31]. The most common genetic variants associated with LQTS are transmitted in an autosomal dominant pattern. However, mutations following an autosomal recessive pattern have also been described. The main autosomal recessive form of LQTS is called Jervell and Lange-Nielsen syndrome [32]. This is a rare form of the LQTS caused by homozygous or compound heterozygous mutations in *KCNQ1* or *KCNE1*. This form of LQTS is also characterized by systemic manifestations. The main clinical manifestations of the Jervell and Lange-Nielsen syndrome include severely prolonged QTc-intervals, life-threatening arrhythmias, and sensorineural deafness. Homozygous or compound heterozygous mutations in the absence of deafness have also been reported and are referred to as autosomal recessive LQTS [32]. Two other LQTS variants, specifically Anderson-Tawil syndrome (LQTS7) and Timothy syndrome (LQTS8), are characterized by extracardiac phenotypes. Anderson-Tawil syndrome is caused by loss-of-function mutations in the potassium channel gene *KCNJ2*. Its main clinical features include QT-interval prolongation, facial dysmorphism, and hypokalaemic periodic paralysis [32]. Timothy syndrome represents the most severe variant of LQTS. It has a high mortality rate, and, of note, it is the only LQTS form in which death is caused by extracardiac phenotypes. Its main clinical features include marked QT-interval prolongation, severe ventricular arrhythmias, congenital heart defects, atrio-ventricular block, syndactyly, autism, malignant hypoglycaemia, and immune system abnormalities. It is primarily caused by a heterozygous G406R mutation in the L-type calcium channel gene *CACNA1C* [32].

Approximately 20% of the families meeting clinical diagnostic criteria for LQTS do not have detectable pathogenic variants in one of the above-mentioned genes. In this regard, recent studies have identified novel LQTS-related genetic variants. An interesting study by Kauferstein and colleagues [33] describes the case of an 18-year-old female patient with symptomatic LQTS who carried a mutation in the cardiac ryanodine receptor (*RyR2*) gene [33]. Interestingly, in principle, the patient was referred as having “genotype-negative” LQTS since no mutations were identified in any of the major LQTS-related genes (*SCN5A*, *KCNH2*, *KCNQ1*, *KCNE1*, *KCNE2*, and *KCNJ2*) [33]. Similarly, Altmann and colleagues identified recessively inherited *TRDN* frameshift mutations in a 10-year-old female patient by analysing a number of patients with genetically elusive LQTS. The *TRDN* gene encodes the cardiac-specific isoform of triadin, a protein associated with the release of calcium ions from the sarcoplasmic reticulum. Structural or functional disruption of this cardiac calcium release unit can lead to significant ventricular arrhythmias [34]. Mutations in the *TRDN* gene can now be considered a novel underlying genetic cause for recessively inherited LQTS [34]. Additionally, a recent study by Riuro and colleagues [35] identified a novel *SCN1Bb* gene mutation (β1bP213T) in an 8-year-old boy who was clinically diagnosed with LQTS without mutations in the common LQTS-related genes. The *SCN1Bb* gene encodes the sodium channel β-1 subunit. The authors show that this new mutation enhances the late sodium current. In detail, the β1bP213T mutation subtly alters Na_v_1.5 function by shifting the window current, accelerating recovery from inactivation, and decreasing the slow inactivation rate. Additionally, by using HL-1 cells, they show that the action potential duration significantly increases when the mutant β1b is overexpressed in comparison with the wild type protein [35].

### Short-QT syndrome

A rare congenital cardiac channelopathy which has been recently described is the short-QT syndrome (SQTS), which is characterized by a very short QT interval and considerable susceptibility to atrial and ventricular fibrillation in the absence of structural heart disease [36, 37]. Specifically, SQTS is diagnosed in the presence of a QTc < 340 ms [38]. However, SQTS should be considered also in the presence of a QTc ≤ 360 ms and one or more of the following conditions: a confirmed pathogenic mutation, a family history of SQTS, a family history of SD at age < 40 years, and/or survival of a ventricular fibrillation/ventricular tachycardia episode in the absence of heart disease [38]. Among the major channelopathies, SQTS may be considered the most severe; cardiac arrest and SCD are the most common manifestations [15]. Although SQTS has an autosomal-dominant inheritance pattern and high penetrance [16], a study by Mazzanti and colleagues suggests that age and sex are associated with susceptibility to SQTS. In particular, cardiac arrest resulting from an abbreviated repolarization was predominantly observed among males and mainly observed either during the first year of life or between 20 and 40 years of age [39]. To date, six main genetic potassium and calcium channel variants have been linked to SQTS. These include *KCNH2*, *KCNQ1*, *KCNJ2*, *CACNA1C*, *CACNA2D1,* and *CACNB2* (Table 2). Gain-of-function mutations in the potassium channel genes (*KCNH2*, *KCNQ1*, and *KCNJ2*) lead to QT interval shortening through increases in repolarizing currents, whereas loss-of-function mutations in the calcium channel genes (*CACNA1C*, *CACNA2D1,* and *CACNB2*) shorten the action potential through decreases in depolarizing currents [32]. Due to the few families available for investigation, no genotype-phenotype correlation studies have been performed. Since it is clear that identical SQT-associated mutations may yield a varied phenotype, recent guidelines suggest making a patient-oriented rather than a family-oriented clinical decision [29]. Treatment decisions in high-probability cases of SQTS are not influenced by genetic findings [29].

**Table 2:** Pathogenic gene mutations associated with SQTS variants

Mutated gene	Encoded protein	Functional alteration	SQTS variant
*KCNH2*	α-subunit of the voltage-gated potassium channel that mediates the rapidly activating component of the delayed rectifying potassium current (I_Kr_)	Gain-of-function mutation that leads to an increased potassium current and shortening of the action potential [36, 129]	SQTS1
*KCNQ1*	α-subunit of the voltage-gated potassium channel that mediates the slow component of the delayed rectifier potassium current (I_Ks_)	Increased repolarizing current [112]	SQTS2
*KCNJ2*	Inward rectifier potassium channel Kir2.1 (I_K1_)	Gain-of-function mutation that leads to an increase in the outward I_K1_ current and acceleration of the final phase of repolarization [130]	SQTS3
*CACNAC1C*	α1-subunit of the L-type calcium channel	Loss-of-function mutation leading to a reduction in the depolarizing current [54]	SQTS4
*CACNB2*	β2-subunit of the L-type calcium channel	Loss-of-function mutation leading to a reduction in the depolarizing current [54]	SQTS5
*CACNA2D1*	α-2/δ subunit of the L-type calcium channel	Loss-of-function mutation leading to a reduction in the depolarizing current [131]	SQTS6

### Brugada syndrome

Brugada syndrome (BrS) is another important congenital cardiac channelopathy. BrS is a hereditary disease clinically characterized by right ventricular conduction delay and ST-segment elevation in the anterior right precordial leads. Syncope is one of the main clinical manifestations; individuals with BrS develop a monomorphic ventricular tachycardia that may precipitate during sleep, rest or fever. Premature SCD may occur due to ventricular fibrillation [31, 40]. BrS displays an autosomal dominant pattern of inheritance with incomplete penetrance. BrS penetrance is age- and sex-related; most lethal events have been reported among men after the fourth decade of age [41–44]. The first genetic alteration to be associated with BrS was a loss-of-function mutation affecting the *SCN5A* gene [45]. This finding was rapidly followed by several other reports. Currently, more than 450 pathogenic variants have been identified in 24 genes encoding sodium, potassium, and calcium channels or associated proteins [16, 46]. *SCN5A* genetic variants accounts for the vast majority (75%) of BrS genotype-positive cases. However, the yield of *SCN5A* genetic testing for robust clinical cases of BrS is approximately 25% [29]. *SCN5A* mutations accounting for BrS have also been described as responsible for SCD in children [44]. *SCN5A* mutations cause a loss of function in the sodium current. It has been reported that the reduction in sodium current amplitude may be due to either decreased expression of the sodium channel protein (Na_v_1.5) in the sarcolemma, expression of non-functional channels, or changes in gating properties (delayed activation, earlier inactivation, faster inactivation, enhanced slow inactivation, or delayed recovery from inactivation) [41]. Mutations affecting the genes encoding the β-subunits of the Na_v_1.5 protein (i.e., *SCN1B, SCN2B,* and *SCN3B*) have also been described as causes of BrS. These genetic variants may have different effects on the sodium current, by direct action on ion conductance or by interference with sodium channel trafficking [47, 48]. Indeed, impairment of Na_v_1.5 trafficking has been described as an additional cause of sodium current reduction. In this regard, other genetic variants have been identified. For instance, mutations in the RAN guanine nucleotide release factor (*RANGRF*) gene, which encodes MOG1-a nuclear import/export protein recently described as a new partner of Na_v_1.5 [49]-have been reported to impair the sodium current by altering Na_v_1.5 trafficking [50]. A similar outcome has been associated with genetic variants of the *SLAMP* gene, which encodes a sarcolemmal membrane-associated protein whose function at the T-tubule level is still unknown [49]. Genetic variants of the glycerol-3-phosphate dehydrogenase 1-like (*GPD1-L*) gene have also been described as a possible cause of BrS [51]. Although GPD1-L is not an ion channel itself, an altered form of this protein may impair trafficking of the α-subunit of the Na_v_1.5 cardiac sodium channel to the surface membrane, thus decreasing the sodium current. An interesting work by London and colleagues demonstrates that GPD1-L mutations may affect protein density and the number of functional sodium channels. However, the actual mechanism by which GPD1-L genetic variants alter sodium channel membrane expression remains to be established since no interaction between the two proteins has been identified to date. Other genetic variants affecting the sodium current include those related to the gene encoding the desmosomal protein plakophilin-2 (*PKP2*) [52] and the transient receptor potential melastatin protein number 4 (*TRPM4*), a calcium-activated nonselective cation channel [53], whose alteration may affect membrane resting potential and consequently alter sodium availability. Although sodium current alteration is considered a leading cause of BrS, calcium current impairment has also been reported to play a critical role in this pathology (4–5% of BrS patients). BrS-related genetic variants have been identified among genes encoding different subunits of the L-type calcium channel, namely, *CACNA1C*, *CACNB2b*, and *CACNA2D1* [54–56]. Potassium currents have also been considered important determinants. In some cases of BrS, gain-of-function mutations have been identified among genes encoding channels that conduct outward potassium current, i.e., *KCND3*, *KCNE*3, *KCNE5,* and *KCNJ8* [41].

Collectively*, SCN5A* is the main gene associated with BrS. *CACNA1C* genetic variants have also been frequently described (4–5%), whereas the remaining genes are the likely cause in less than 1% of disease manifestations (per gene). Known BrS-susceptibility genes can only partially explain the clinically diagnosed cases; therefore, many patients (65–70%) remain “genetically unresolved” [16, 46].

### Catecholaminergic polymorphic ventricular tachycardia

Catecholaminergic polymorphic ventricular tachycardia (CPVT) is a pathological condition in which a ventricular arrhythmia may be triggered by intense physical exercise or acute emotional stress. Typical clinical manifestations of CPVT include dizziness and syncope. However, ventricular arrhythmia may degenerate into rapid polymorphic ventricular tachycardia and ventricular fibrillation, leading to SCD [16, 57]. Since individuals with CPVT have normal resting electrocardiograms, diagnosis may be difficult. CPVT is characterized by both autosomal dominant and autosomal recessive (less frequent) patterns of inheritance. As reported in Table 3, CPVT is caused by mutations in genes encoding ion channels or calcium-handling proteins that primarily affect the electrical activity of the heart. In particular, the first genetic alteration associated with CPVT was identified in the *RYR2* gene in 2001 [58]. Pathogenic variants of the *RYR2* gene account for more than 50% of CPVT cases (CPVT1). For instance, in a recent review, Sumitomo reported data obtained from a cohort of patients from his institution. Specifically, he described that 79% of the observed CPVT cases were related to an anomaly in the *RyR2* gene. He also reported that SD was observed in approximately 10% of these patients, without any sex-related difference. The inheritance of CPVT1 is autosomal-dominant [57]. CPVT2, which is the second most common subtype of CPVT, is related to mutations in the gene encoding the calsequestrin protein (*CASQ2* gene). According to the analysis by Sumitomo, the rate of SCD observed for CPVT2 was higher than for CPVT1. CPVT2 shows an autosomal recessive pattern of inheritance, although autosomal dominant mutations are also reported [57]. Overall, genetic screening permits identification of mutations in up to 65% of patients with a clinical diagnosis [29]. Other CVPT-susceptibility genes include *CALM* (encoding the protein calmodulin) and *TRDN* (encoding triadin), responsible for CPVT4 and CPVT5, respectively (Table 3). However, as shown in Table 3, genetic causes of CPVT remain to be elucidated. For instance, CPVT3 was reported in a family carrying a 7p22-p14 chromosome anomaly. However, the responsible gene has not been identified to date. Interestingly, a recent article by Devalla and colleagues described a new life-threatening arrhythmia related to trans-2,3-enoyl-CoA reductase-like (*TECRL*) genetic variants [59]. *TECRL* encodes a protein involved in fatty acid and lipid metabolism, with a function probably related to catalysis of redox reactions [59]. Homozygous *TECRL* mutations were identified by using whole-exome sequencing in patients from three different families with life-threatening arrhythmias and high risk of SCD. Interestingly, this recessive form of inherited arrhythmia has a clinical phenotype with overlapping features of both LQTS and CPVT. Perturbations in physiological levels of lipids/fatty acids and metabolic functions can have direct consequences on ion channels and calcium-handling proteins [59]. However, further investigations are needed to fully clarify the role of *TECRL* in lipid metabolism and how its genetic variants may be linked to cardiac arrhythmias [59]. Overall, this study highlights the significant contribution of genomic technologies to the understanding of underlying causes of SCD.

**Table 3:** Pathogenic gene mutations associated with CPVT variants

Mutated gene	Encoded protein	Functional alteration	CPVT variant
*RyR2*	Cardiac ryanodine receptor involved in calcium release from the sarcoplasmic reticulum, mediating excitation–contraction coupling	Increase of spontaneous intracellular calcium, oscillations, delayed after-depolarizations, and spatial heterogeneity of repolarization, leading to polymorphic ventricular tachycardia [31, 57]	CPVT1
*CASQ2*	Calsequestrin, a regulatory protein associated with the ryanodine receptor	Dysregulation of calcium homeostasis [57, 132]	CPVT2
*7p22-p14 chromosome anomaly* [133]	?	?	CPVT3
*CALM*	Calmodulin	Calcium overload [57, 134, 135]	CPVT4
*TRDN*	Triadin, a protein that connects calsequestrin to the ryanodine receptor stabilizing the calcium channel	Diastolic calcium leak and calcium overload in myocytes [57]	CPVT5

## Cardiomyopathies

Along with cardiac channelopathies, cardiomyopathies may be considered leading causes of SCD. Cardiomyopathy often results in the heart failure syndrome, with a consistent number of systemic manifestations. Early clinical investigations have recognized familial transmission for many cardiomyopathies, suggesting a genetic basis of this disease. This hypothesis now has been widely confirmed by intensive research. Accordingly, many cardiomyopathies are currently recognized as monogenic disorders [18, 60].

### Hypertrophic cardiomyopathy

Hypertrophic cardiomyopathy (HCM) is one of the most frequently observed inherited cardiomyopathies (incidence of approximately 0.2%). Mutations in genes encoding cardiac sarcomeric proteins are the main causes of HCM. The main pathological hallmark of HCM is hypertrophy of the left ventricle. Clinical manifestations of HCM may range from dyspnoea and/or syncope to SCD due to ventricular fibrillation [61]. HCM has an autosomal dominant pattern of inheritance. The main genes (and encoded proteins) involved include the following: *MYH7* (β-myosin heavy chain), *TPM*, (α-tropomyosin), *TNNT2* (cardiac troponin T), *MYBPC3* (cardiac myosin binding protein), *MYL2* (myosin regulatory light chain), *MYL3* (myosin essential light chain), *TNNI3* (cardiac troponin I), *ACTC1* (cardiac α-actin), *TNNC1* (cardiac troponin C), *MYH6* (α- myosin heavy chain), and *PRKAG2* (protein kinase A, γ-subunit of AMP-activated protein kinase) [18, 61]. In addition to the described genetic alterations, novel genetic variants are now emerging, with contributions to phenotypes with different degrees of severity [62]. Disease-causing mutations in *MYH7* and *MYBPC3* are the most common. Each of these mutations accounts for one-quarter to one-third of all cases, with the remaining HCM genes each account for 1 to 5% or less [29].

Other genetic variants are associated with HCM. These include mutations in the myozenin 2 (*MYOZ2*) gene, encoding a Z-disc protein [63], and the *ACTN2* gene, encoding α-actinin-2, the major component of the Z-disc. Although such mutations are uncommon and need to be further investigated, they support the hypothesis that disruption of Z-disc proteins can lead to HCM [64].

It has been reported [29] that the diagnostic yield of sarcomere gene testing (comprising up to 9 genes) in clinical cases of familial HCM is typically approximately 60%. The yield depends on patient selection and decreases to approximately 30% in sporadic disease. Approximately 5% of cases have two or more variants (compound or double heterozygotes), although in many cases, at least one of the variants is of uncertain significance [29].

### Dilated cardiomyopathy

Dilated cardiomyopathy (DCM) is characterized by the presence of left ventricular dilatation and contractile dysfunction with heart failure [65]. Ventricular and supraventricular arrhythmias, conduction system abnormalities, and thromboembolism are often observed. In later stages of the disease, SCD may also occur. DCM can be classified by cause as familial, primary without a family history, or secondary (associated with or caused by other conditions). The familial form is considered to be responsible for at least 20% of idiopathic cases [66]. This type of DCM has an autosomal dominant pattern of inheritance. X-linked, autosomal recessive, and mitochondrial inheritance patterns have also been identified, but they are uncommon [65]. DCM-related mutations have been identified in genes encoding a wide range of proteins, particularly those affecting sarcomere function, electrolyte (calcium, sodium) homeostasis, and nonsarcomere structure [18]. In pure dilated cardiomyopathy, the screening yield for a large number of genes is approximately 20% [67]. The most frequently identified mutations involve the *TTN* gene, which encodes titin, the main component of the sarcomere [68]. It has been reported that *TTN* mutations may account for up to 25% of all cases of autosomal dominant DCM [65]. Some genetic variants of the *TTN* gene have been observed both in DCM patients and in healthy controls, suggesting the presence of polymorphisms unrelated to DCM. For instance, a screen of the *TTN* gene in a cohort of 120 genetically unrelated patients with DCM identified seven sequence variations leading to amino acid replacements. Three of the mutations were also found among the healthy controls [69]. Another report described the screening of 312 DCM patients in which *TTN* truncating variants, most commonly found at the A band, were found in 18% of sporadic DCM, 25% of familial DCM, and 3% of healthy controls [70]. Even truncating variants in the Z-band and I-band are highly present in controls, while variants affecting the M-band are mostly associated with a neuromuscular phenotype [70]. In addition to the above-mentioned genetic variants, more than 60,000 missense variants have also been identified, in the 1000 Genomes Project [70–72]. However, this frequency is far above the expected frequency of disease causing mutations for the *TTN* gene [71]. Golbus and colleagues [73] analyzed the data of the 1000 Genomes Project and found a high frequency of predicted pathogenic protein altering variation in *TTN*. They suggested that many of these variants could be either benign or insufficient on their own to cause disease, but could act as modifiers in genetically susceptible hosts [71]. However, even when stringent bioinformatic and segregation criteria are used, the analysis of *TTN* missense variants contribution to DCM phenotype is very challenging. For instance, the interesting analysis carried out by Begay and colleagues [71] identified variants bioinformatically classified as “severe” in 12.6% (44/348) of *TTN* missense variants and 27.6% (37/134) of DCM subjects. They reported that 5 of 9 families with *TTN* variants classified as “severe” demonstrated incompatible segregation with the affected phenotype, implying a significant false-positive rate from the bioinformatics analysis alone. Four families harboured 5 “severe” *TTN* variants that segregated with the DCM phenotype, and 28 probands had “severe” variants that could not be assessed by segregation. Therefore, in contrast to *TTN* truncated variants, pathogenic *TTN* missense variants are not easy to resolve and likely contribute to a small fraction of DCM cases [70, 71]. Of note, the same authors reported that the distribution of bioinformatically “severe” *TTN* missense variants across titin domains was non-random and similar to what has been shown previously with *TTN* truncation variants that were overrepresented in the A-band region of titin.

DCM-related genetic variants have also been recently found in other genes encoding sarcomere proteins, namely α-cardiac actin, α-tropomyosin, cardiac troponin T, I, and C, β- and α-myosin heavy chains, myosin binding protein C, and α-actinin-2 [65].Of note, such genetic variants have also been observed in HCM. Additionally, hypertrophic and dilated phenotypes may overlap in some families, emphasizing that definition of the morphological features of cardiomyopathy may also have a key role in diagnosis. Among the mutations impairing electrolyte homeostasis, *PLN* gene mutations may contribute to the DCM phenotype [74]. *PLN* encodes phospholamban, a protein that modulates calcium uptake by the calcium-transporting ATPase of the sarcoplasmic reticulum (SERCA2a). Mutations in the *SCN5A* gene have similarly been implicated in DCM. However, this gene has also been associated with other cardiac diseases that may cause SD, confirming that examination of morphological features may be necessary to clinically characterize DCM. Among structural proteins, mutations in the *LMNA* gene encoding the lamin-A and -C nuclear envelope proteins may account for up to 5% of all autosomal dominant DCM cases [65, 75]. These proteins are ubiquitously expressed and play key roles in the maintenance of proper nuclear structure. Alterations of laminin-A and -C proteins may cause dilated cardiomyopathy, atrioventricular block, and both atrial and ventricular fibrillation [65]. *LMNA* mutations are highly predictive for progressive conduction disease and SD risk. Although clinical identification of affected subjects at high risk of SCD is quite difficult, the identification of *LMNA* mutations has recognized prognostic value for the diagnosis of DCM. Overall, the genetic heterogeneity of DCM and the overlap of mutations with other cardiac diseases have prevented the assessment of a direct correlation between genetic features and this specific pathology. As reported above, the same mutation may be the cause of DCM or HCM in different unrelated subjects. However, heart function and morphology may be affected differently, suggesting that factors other than genetic ones (e.g., environment) may influence the phenotypic expression of a primary cardiomyopathy.

### Restrictive cardiomyopathy

Restrictive cardiomyopathy (RCM) is another important cardiomyopathy characterized by ventricular stiffness, which results in severe diastolic dysfunction and restrictive filling with elevated cardiac filling pressures and dilated atria. Hypertrophy is typically absent [76]. The diagnosis is mainly based on functional findings. Restrictive physiology also occurs in HCM and DCM, thus contributing to difficult disease classification. Furthermore, the genetic features of RCM may overlap with DCM and HCM. RCM-associated mutations have been reported in four genes that encode key sarcomeric proteins/myofilaments (*MYH7*, *TNNT2*, *TNNI3*, and ACTC). *MYH7*- and *TNNI3*-mediated RCM may each account for approximately 5% of cases [29]. In fact, some authors consider RCM to be part of a phenotypic spectrum of HCM with limited hypertrophy and restrictive physiology [18]. However, as a distinct genetic cardiomyopathy, RCM is transmitted in an autosomal-dominant pattern, although autosomal recessive and X-linked patterns of inheritance have been described [77]. Over the past 10 years, the introduction of high-throughput sequencing methods (also called next-generation sequencing, NGS) [78] has dramatically improved the identification of RCM susceptibility genes. An interesting study by Kostareva and colleagues [79] describes the analysis of 24 cases of RCM performed by using an NGS approach. The authors identified a broad spectrum of RCM-associated variants in approximately 54% of patients, in line with data obtained from another study that found genotype-positive cases in 19 (60%) of 32 unrelated RCM patients [80]. Of note, the former study confirmed the key role of sarcomeric proteins in the development of RCM and extended the spectrum of pathogenic mutations to genes encoding structural and cytoskeletal proteins. Despite the small number of patients included in the study, the results appeared to disclose a multifactorial nature for RCM, which, according to this view, could be triggered by a combination of multiple mutations rather than a single disease-causing mutation. This condition may influence the clinical manifestations and the incidence of SD. In line with these observations, recent findings have corroborated the hypothesis that factors other than pathogenic genetic variants may modulate disease phenotypes in primary cardiomyopathies, with modifier genes are emerging as key players. In detail, the term “modifier genes” refers to genetic variants that are not directly responsible for the disease but that can influence the phenotypic expression of the primary mutation [81]. Recent studies have demonstrated that mutations in genes related to the renin-angiotensin pathway may influence the clinical phenotypes of HCM, DCM, and RCM. Mutations in the angiotensin I-converting enzyme, angiotensinogen (AGT), and AGT Receptor type 1 seem to be specifically involved [81–84].

### Arrhythmogenic right ventricular cardiomyopathy

Arrhythmogenic right ventricular cardiomyopathy (ARVC) is a rare but increasingly recognized condition characterized by the replacement of myocytes with adipose and fibrous tissue, leading to right ventricular failure, arrhythmias, and SCD [85]. Because of fatty infiltration, ARVC is also called fat cardiomyopathy. ARVC is one of the most important causes of SCD among young people, especially athletes [86]. The rate of SD has been estimated at approximately 2.5% per year [87]. There is familial evidence of ARVC in more than 60% of patients [86]. Although most patients show an autosomal dominant pattern of inheritance [88], two recessive modes of ARVC inheritance have been recently identified. The first to be reported was the “Naxos disease”, a cardiocutaneous syndrome caused by homozygous mutations in genes encoding the cell adhesion proteins plakoglobin and desmoplakin. A variant of Naxos disease (overlapping clinically with DCM) with predominant left ventricular involvement was reported a few years later under the name of “Carvajal syndrome.” All patients with these recessively inherited conditions had a peculiar cutaneous phenotype (woolly hair and a palmoplantar keratoderma) from infancy and developed ARVC by adolescence [89]. The genetic background of ARVC has been extensively studied; many causative genes have been identified during the last 10 years. The first genetic mutations associated with ARVC were found in the desmosomal genes [90]. Desmosomes are indispensable protein complexes involved in electrical conduction and mechanical contraction of cardiomyocytes. They are composed of five proteins: junctional plakoglobin (encoded by *JUP*), plakophilin-2 (encoded by *PKP2*), desmoplakin (encoded by *DSP*), desmoglein-2 (encoded by *DSG2*), and desmocollin-2 (encoded by *DSC2*). Specifically, *JUP* was the first gene whose mutations were associated with ARVC [90]. This finding was rapidly followed by the identification of mutations involving other desmosomal genes: *DSP* in 2002, *PKP2* in 2004, and both *DSG2* and *DSC2* in 2006 [91–94]. Among them, the most frequently observed mutations occur in the *PKP2* gene. In western countries, more than 70% of ARVC patients with desmosomal gene mutations carry *PKP2* mutations [95]. The yield of genotyping is variable; the occurrence of founder mutations may increase the yield of genotyping in selected regions. Interpretation of the pathogenic role of missense mutations in genes encoding desmosomal proteins is complicated by evidence that fewer than 16% of control individuals harbour missense variants that would meet clinical criteria for a positive genetic test result [32]. Overall, desmosomal gene mutations can be identified in approximately 50% of ARVC patients [96]. Other genetic mutations in extra-desmosomal proteins, which indirectly affect intercalated disc function, have also been reported as causative genes for ARVC (Table 4). In this regard, mutations in the *TMEM43* gene may cause a lethal, fully penetrant, sex-influenced, autosomal dominant disorder [97]. During recent years, research has been focused mainly on the understanding of genetic factors underlying altered expression of desmosomal and extra-desmosomal proteins, including those involved in downstream signalling pathways. However, clinical evidence of desmosomal dysfunction in ARVC patients not carrying known genetic mutations suggests that other mechanisms, likely epigenetic factors, may also lead to the pathogenesis of ARVC. Among those, alterations in the Hippo pathway [98] and microRNAs [99] have been recently identified as potential regulators of arrhythmogenic cardiomyopathy. Among the molecular mechanisms implicated in the pathogenesis of ARVC, abnormal intracellular calcium handling is also involved. More recently, increasing attention has been given to calcium sensitive signalling proteins, which play a leading role in electrical and structural remodelling of the heart in the setting of ARVC. Calcium impairment with consequent activation of calmodulin-dependent kinase II and calcineurin can also directly affect the integrity of intercalated disk structure contributing to broad phenotypic variability among ARVC patients with or without known genetic mutations [100].

**Table 4:** Pathogenic gene mutations associated with ARVC

Mutated gene	Encoded protein	Functional alteration	Genotype
*TGF-β3*	Transforming growth factor β	Overexpression of TGF-β protein, leading to myocardial fibrosis [136, 137]	ARVC1
*RYR2*	Ryanodine receptor	Mutations appear to unblock the channel, resulting in hyperactivation/hypersensitization [138, 139]	ARVC2
*Unknown, chromosome 14q23–q24 (locus D14S42)*	Unknown	Unknown [140]	ARVC3
*TTN*	Titin	Increased vulnerability to proteolysis and degradation [141]	ARVC4
*TMEM43*	Transmembrane protein 43, a nuclear membrane organizer	It has been hypothesized that TMEM43 is a member of an adipogenic pathway regulated by PPARγ. Therefore, its dysregulation may impact the entire pathway, thus explaining the fibrofatty replacement of the myocardium in ARVC patients [97]	ARVC5
*Unknown, chromosome 10p12-p14*	Unknown	Unknown [142]	ARVC6
*DES*	Desmin, the intermediate filament protein expressed by cardiac cells	Aggresome formation [143]	ARVC7
*DSP*	Desmoplakin	Altered binding to plakoglobin and plakophilin [92]	ARVC8
*PKP2*	Plakophilin-2	Disruption of functionally important domains of the PKP2 protein [94, 144]	ARVC9
*DSG2*	Desmoglein-2	Possible change in affinity and abolition of adhesive capacity [91]	ARVC10
*DSC2*	Desmocollin-2	Frameshifts and premature termination codons, leading to a completely nonfunctional mutant protein with no adhesive capacity [93]	ARVC11
*JUP*	Junctional plakoglobin	Increased expression of adipogenic factors [90]	ARVC12
*PLN*	Phospholamban	It has been hypothesized that mutant phospholamban may impair SERCA2a activity, leading to calcium homeostasis impairment, which in turn may result in desmosomal disassembly [145]	Others
*LMNA*	Lamin A/C	Increase in nuclear deformation, fragmentation of chromatin, and abnormal mechanotransduction, leading to impaired ability of the cell and nuclei to resist mechanical stress [146]	Others
*SCN5A*	α-subunit of the cardiac sodium channel Na_v_1.5	Loss of function [147]	Others
*CTNNA3*	α-T-catenin, which binds to plakophilins, participating in adhesion between cardiomyocytes	Impaired interaction with β-catenin and increased dimerization potential [148]	Others

### Left ventricular non-compaction

Left ventricular non-compaction cardiomyopathy (LVNC) is characterized by segmental thickening of the left ventricular wall with a thin, compact epicardial layer as well as an excessively thickened endocardial layer with prominent, deep intertrabecular recesses [101]. During the last 25 years, LVNC has gained increased attention. However, its classification is still a matter of debate. In 2006, the American Heart Association recognized LVNC as a primary genetic cardiomyopathy [102]. However, the European Society of Cardiology classified LVNC as an “unclassified cardiomyopathy” [77] because it is still unclear whether it represents a distinct cardiomyopathy or merely a phenotypic trait common to other cardiomyopathies. Familial disease has been estimated to occur in approximately 18–50% of adults diagnosed with LVNC, mostly consistent with an autosomal dominant mode of inheritance [101]. LVNC-related mutations have been mostly identified in sarcomeric genes as observed in other cardiomyopathies such as HCM and DCM, thereby suggesting a shared genetic susceptibility [101]. Recently, a novel *RYR2* genetic variant with a phenotype overlapping atypical CPVT and LVNC was identified in a multigenerational family [103]. Although several LVNC-susceptibility genes have been identified, none predominates. Moreover, evaluations of large populations have not been reported [29]. A relatively small percentage of patients (approximately 15 to 20%) with LVNC have known genetic mutations, although larger panels of genes may proportionately increase the yield [29]. For instance, a work by Klaassen and colleagues reported a 17% mutation rate when considering 6 genes among 63 unrelated adult index cases [29, 104]. More recently, Hoedemaekers and colleagues identified mutations in 23 of 56 index cases by using a 17-gene panel [29, 101]. Due to the low positive genetic test rate among index cases, the utility of testing for definitive diagnosis and care of these cases is limited [29].

## A focus on *CAV3* genetic variants

Caveolins are the main constituents of the caveolae, which are small (50–100 nm) plasma invaginations composed of cholesterol and other lipids. These structures contain several protein complexes involved in numerous cellular processes such as vesicular trafficking, protein targeting, second messenger signalling, cholesterol homeostasis, mechanosensing and survival responses to stressful stimuli [22]. In particular, caveolin-3, encoded by the *CAV3* gene, is one of the three major forms of caveolins selectively expressed within the heart. Many cardiac ion channels and transporters, including L-type calcium channels, potassium channels, sodium channels, the sodium/potassium ATPase, and the sodium/calcium exchanger have been found within caveolae. This specific subcellular localization is critical for the function of these components since it allows integration within macromolecular signalling complexes, thus ensuring precise regulation.

*CAV3* genetic variants have been associated with a variety of diseases, including rippling muscle disease, limb-girdle-muscle dystrophy, muscular dystrophy, and cardiac-related disorders [105]. The first *CAV3* mutation associated with HCM was identified in a case involving siblings [24]. According to research by Hayashi and colleagues, these patients showed an atypical clinical phenotype, with mild *CAV3*-induced HCM characterized by high ECG voltage and suggestive diastolic dysfunction with elevated LV end-diastolic pressure. Noteworthy, the father of the siblings, an obligate carrier of the *CAV3* mutation, died suddenly at the age of 41. Interestingly, the study reported that the HCM-related mutation occurred at the same codon as observed for limb-girdle-muscle dystrophy. Analysis of the cellular effect of the *CAV3* mutation revealed that the mutated caveolin-3 protein had reduced surface expression compared to the wild type protein. This change appeared to be relatively mild in the case of the HCM-related mutant, indicating that the dysfunctions induced by *CAV3* mutations are of differing severity in the two pathologies. The greater severity of the muscular-dystrophy-related mutation explains the cardiac involvement (DCM) observed in patients with this disease. In contrast, the presence of HCM mutations does not necessarily imply muscular involvement; in fact, the siblings did not exhibit any muscular disorder. This may be because caveolin functions may be differently regulated in cardiac and muscle tissues. A study by Cagliani and colleagues [106] also supports this observation. The authors identified a heterozygous 3-bp microdeletion (328–330 del) in individuals affected by skeletal muscle diseases such as limb-girdle muscular dystrophy type 1C, rippling muscle disease, and both sporadic and familial forms of hyperCKemia. The mutation resulted in the loss of a phenylalanine (Phe97del) in the transmembrane domain, leading to a severe caveolin-3 deficiency and caveolar disorganization in the skeletal muscle. Of note, caveolin-3 was expressed in the myocardium to a degree corresponding to approximately 60% of that of control individuals and was correctly localized at the myocardial cell membranes, with preservation of cardiac myofibre caveolar structures [106]. Thus, the same mutation may lead to different phenotypes. However, clear evidence in the literature has shown that muscular and cardiac dysfunctions related to *CAV3* mutations may also coexist [107, 108]. This observation emphasizes the importance of carefully monitoring myopathic patients carrying *CAV3* mutations for cardiac involvement. It is worthwhile to report the case of a German family with rippling muscle disease in which two members died suddenly of possible cardiac arrhythmias. Autopsies of one of these patients revealed a non-obstructive cardiomyopathy [107, 109].

*CAV3* mutations have also been related to LQTS. In 2006, Vatta and colleagues reported, for the first time, a relationship between *CAV3* mutations and LQTS [25]. In particular, they demonstrated that caveolin-3 directly modified cardiac sodium channel kinetics. Therefore, *CAV3* mutations, by altering sodium currents, have been directly associated with the pathogenesis of the LQTS variant LQTS9. In particular, it has been shown that coexpression of the caveolin-3 mutation and the *SCN5A* gene results in an increased late sodium current. Cronck and colleagues [5] identified three *CAV3* mutations (V14 L, T78 M, and L79R) among a population of 133 infants who died from SIDS. Voltage clamp analysis revealed that all the three mutations were responsible for a fivefold increase in the late sodium current. The mechanism underlying this finding was recently clarified. Cheng and colleagues reported that the increase in cardiac sodium current related to caveolin-3 mutations may be reversed by the neural nitric oxide synthase (nNOS) inhibitor L-NG-monomethyl arginine citrate. The authors propose a mechanism whereby wild type caveolin-3 negatively regulates nNOS. A *CAV3* mutation (F97C) may cause the loss of this inhibitory effect, thereby accentuating local NO and increasing the late sodium current via direct S-nitrosylation of the α-subunit of the sodium channel Na_v_1.5. This, in turn, may significantly prolong the action potential duration, as observed in the LQTS clinical electrophysiological phenotype [110].

**Fig. 1:**
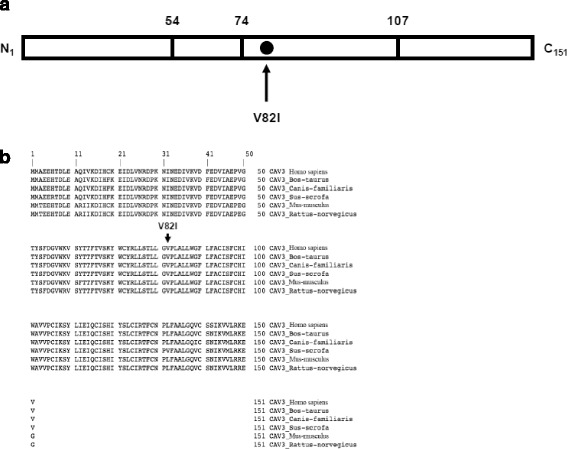
Caveolin-3 topological domains (**a**) and localization of the V82I variation (**b**) identified in a LQTS patient. The figure has been completely adopted from [22]

**Fig. 2:**
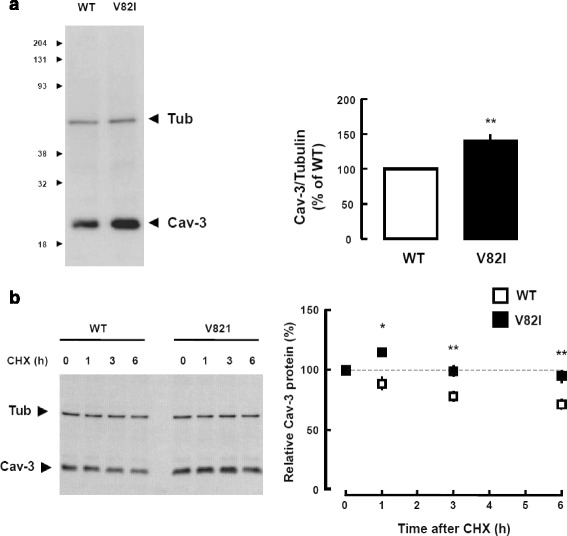
Expression levels and stability of caveolin-3 wild type and caveolin-3 V82I mutant. **a** Recombinant expression of caveolin-3 wild type (Cav-3 WT) and caveolin-3 V82I mutant (Cav-3 V82I) in BHK cells transiently transfected with plasmids expressing either Cav-3 WT or Cav-3 V82I. **b** Evaluation of protein stability. Transfected BHK cells were treated with the eukaryote protein synthesis inhibitor cycloheximide (10 μg/ml) for the indicated length of time. A representative blot is shown on the left of panel b. Levels of residual caveolin-3 at the indicated time points (% of time 0) for Cav-3 WT and Cav-3 V82I mutant are shown on the right. Data are representative of four independent experiments. *, *P* < 0.05 vs WT at the respective time point; **, *P* < 0.01 vs WT at the respective time point. The figure has been completely adopted from [22]

**Fig. 3:**
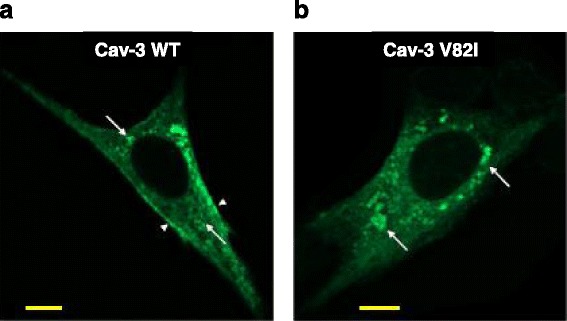
Immunolocalization of caveolin-3 wild type and caveolin-3 V82I mutant. BHK cells were transiently transfected with plasmids expressing caveolin-3 wild type (Cav-3 WT) (**a**) or caveolin-3 V82I mutant (Cav-3 V82I) (**b**) and immunostained with antibodies against caveolin-3 followed by Alexa Fluor 488-conjugated secondary antibodies. Cav-3 V82I was retained intracellularly and not properly targeted to the plasma membrane as with Cav-3 WT (*arrowheads*). Vesicle-like structures stained with caveolin-3 antibodies were also observed, especially for Cav-3 V82I (*arrows*). Scale bar: 10 μm. The figure has been completely adopted from [22]

Recently, a new putative *CAV3* variant (V82I) was identified in a patient with SCD [22]. The study by Lariccia and colleagues reported the case of an adult with suspected LQTS who suddenly died prior to complete comprehensive cardiologic examination. Interestingly, no mutations in the major LQTS-related genes were found, although a novel mutation (V82I) in the *CAV3* gene was identified (Fig. 1). The effect of this mutation has been studied in vitro by using BHK cells. Biochemical analysis of the transiently transfected cell line showed that the caveolin-3 V82I mutant was expressed at higher level than caveolin-3 wild type, as a probable consequence of higher protein stability (Fig. 2). Additionally, confocal microscopy studies revealed that the caveolin-3 V82I mutant tends to accumulate within the cells (Fig. 3), a tendency that has also been observed in a different caveolin-3 mutation [24]. The results also suggest an impairment in the ERK signalling pathway, which is normally regulated by caveolin-3 and influences the cell survival rate. This impairment renders the cells more susceptible to stressful conditions, specifically sub-lethal osmotic stress, thereby suggesting that expression of this novel mutation may be detrimental for the survival response to mechanical stress.

## Conclusions

The knowledge on channelopathies and cardiomyopathies has greatly improved during the last 20 years thanks mainly to advances in the genetic field. Several genes have been demonstrated to be involved in the origin of these cardiac diseases. A proportion of this improved genetic understanding has been applied to the diagnosis and prevention of channelopathies and cardiomyopathies associated with SCD. Genotype studies have become an essential part of clinical diagnosis both to identify asymptomatic individuals who may be at risk of SCD and to unravel genetic alterations when post-mortem examination fails to demonstrate an adequate cause of death. Several mutations related to channelopathies have been reported to occur in genes encoding ion channels or associated proteins. In comparison, for cardiomyopathies, mutations mainly occur in genes encoding structural proteins found in sarcomeres, desmosomes, or the cytoskeleton. Although great achievements have been made in the understanding of genetic contributions to cardiac disorders leading to SCD, a significant number of clinically diagnosed patients still have no recognized genetic cause of disease. Continuous efforts in researching the genetic basis of SCD will be essential for identification of new genetic alterations associated with SCD to improve current diagnostic testing, early prevention, and risk stratification.

## Abbreviations

ARVC: Arrhythmogenic right ventricular cardiomyopathy
BrS: Brugada syndrome
CPVT: Catecholaminergic polymorphic ventricular tachycardia
DCM: Dilated cardiomyopathy
HCM: Hypertrophic cardiomyopathy
LQTS: Long-QT syndrome
LVNC: Left ventricular noncompaction
NGS: Next-generation sequencing
RCM: Restrictive cardiomyopathy
SCD: Sudden cardiac death
SD: Sudden death
SIDS: Sudden infant death syndrome
SQTS: Short-QT syndrome

## References

[1] Basso C, Carturan E, Pilichou K, Rizzo S, Corrado D, Thiene G (2010). Sudden cardiac death with normal heart: molecular autopsy. Cardiovasc Pathol.

[2] Oliva A, Brugada R, D’Aloja E, Boschi I, Partemi S, Brugada J, Pascali VL (2011). State of the art in forensic investigation of sudden cardiac death. Am J Forensic Med Pathol.

[3] Zipes DP, Wellens HJ (1998). Sudden cardiac death. Circulation.

[4] Andreasen C, Refsgaard L, Nielsen JB, Sajadieh A, Winkel BG, Tfelt-Hansen J, Haunso S, Holst AG, Svendsen JH, Olesen MS (2013). Mutations in genes encoding cardiac ion channels previously associated with sudden infant death syndrome (SIDS) are present with high frequency in new exome data. Can J Cardiol.

[5] Cronk LB, Ye B, Kaku T, Tester DJ, Vatta M, Makielski JC, Ackerman MJ (2007). Novel mechanism for sudden infant death syndrome: persistent late sodium current secondary to mutations in caveolin-3. Heart Rhythm.

[6] Ackerman MJ, Siu BL, Sturner WQ, Tester DJ, Valdivia CR, Makielski JC, Towbin JA (2001). Postmortem molecular analysis of SCN5A defects in sudden infant death syndrome. JAMA.

[7] Burke A, Creighton W, Mont E, Li L, Hogan S, Kutys R, Fowler D, Virmani R (2005). Role of SCN5A Y1102 polymorphism in sudden cardiac death in blacks. Circulation.

[8] Plant LD, Bowers PN, Liu Q, Morgan T, Zhang T, State MW, Chen W, Kittles RA, Goldstein SA (2006). A common cardiac sodium channel variant associated with sudden infant death in African Americans, SCN5A S1103Y. J Clin Invest.

[9] Josephson ME (2014). Sudden cardiac arrest. Indian Heart J.

[10] Berdowski J, de Beus MF, Blom M, Bardai A, Bots ML, Doevendans PA, Grobbee DE, Tan HL, Tijssen JG, Koster RW, Mosterd A (2013). Exercise-related out-of-hospital cardiac arrest in the general population: incidence and prognosis. Eur Heart J.

[11] de Vreede-Swagemakers JJ, Gorgels AP, Dubois-Arbouw WI, van Ree JW, Daemen MJ, Houben LG, Wellens HJ (1997). Out-of-hospital cardiac arrest in the 1990’s: a population-based study in the Maastricht area on incidence, characteristics and survival. J Am Coll Cardiol.

[12] Kong MH, Fonarow GC, Peterson ED, Curtis AB, Hernandez AF, Sanders GD, Thomas KL, Hayes DL, Al-Khatib SM (2011). Systematic review of the incidence of sudden cardiac death in the United States. J Am Coll Cardiol.

[13] Risgaard B, Winkel BG, Jabbari R, Behr ER, Ingemann-Hansen O, Thomsen JL, Ottesen GL, Gislason GH, Bundgaard H, Haunso S, Holst AG, Tfelt-Hansen J (2014). Burden of sudden cardiac death in persons aged 1 to 49 years: nationwide study in Denmark. Circ Arrhythm Electrophysiol..

[14] Stecker EC, Reinier K, Marijon E, Narayanan K, Teodorescu C, Uy-Evanado A, Gunson K, Jui J, Chugh SS (2014). Public health burden of sudden cardiac death in the United States. Circ Arrhythm Electrophysiol.

[15] Behere SP, Weindling SN (2015). Inherited arrhythmias: the cardiac channelopathies. Ann Pediatr Cardiol.

[16] Fernandez-Falgueras A, Sarquella-Brugada G, Brugada J, Brugada R, Campuzano O. Cardiac Channelopathies and Sudden Death: Recent Clinical and Genetic Advances. Biology (Basel). 2017;6(1):1–21 10.3390/biology6010007 PMC5372000 28146053

[17] Tang Y, Stahl-Herz J, Sampson BA (2014). Molecular diagnostics of cardiovascular diseases in sudden unexplained death. Cardiovasc Pathol.

[18] Burke MA, Cook SA, Seidman JG, Seidman CE (2016). Clinical and mechanistic insights into the genetics of Cardiomyopathy. J Am Coll Cardiol.

[19] Wexler RK, Elton T, Pleister A, Feldman D (2009). Cardiomyopathy: an overview. Am Fam Physician.

[20] Behr ER, Dalageorgou C, Christiansen M, Syrris P, Hughes S, Tome Esteban MT, Rowland E, Jeffery S, McKenna WJ (2008). Sudden arrhythmic death syndrome: familial evaluation identifies inheritable heart disease in the majority of families. Eur Heart J.

[21] Cohen AW, Hnasko R, Schubert W, Lisanti MP (2004). Role of caveolae and caveolins in health and disease. Physiol Rev.

[22] Lariccia V, Nasti AA, Alessandrini F, Pesaresi M, Gratteri S, Tagliabracci A, Amoroso S (2014). Identification and functional analysis of a new putative caveolin-3 variant found in a patient with sudden unexplained death. J Biomed Sci.

[23] Tang Z, Scherer PE, Okamoto T, Song K, Chu C, Kohtz DS, Nishimoto I, Lodish HF, Lisanti MP (1996). Molecular cloning of caveolin-3, a novel member of the caveolin gene family expressed predominantly in muscle. J Biol Chem.

[24] Hayashi T, Arimura T, Ueda K, Shibata H, Hohda S, Takahashi M, Hori H, Koga Y, Oka N, Imaizumi T, Yasunami M, Kimura A (2004). Identification and functional analysis of a caveolin-3 mutation associated with familial hypertrophic cardiomyopathy. Biochem Biophys Res Commun.

[25] Vatta M, Ackerman MJ, Ye B, Makielski JC, Ughanze EE, Taylor EW, Tester DJ, Balijepalli RC, Foell JD, Li Z, Kamp TJ, Towbin JA (2006). Mutant caveolin-3 induces persistent late sodium current and is associated with long-QT syndrome. Circulation.

[26] Priori SG, Blomstrom-Lundqvist C, Mazzanti A, Blom N, Borggrefe M, Camm J, Elliott PM, Fitzsimons D, Hatala R, Hindricks G, Kirchhof P, Kjeldsen K, Kuck KH, Hernandez-Madrid A, Nikolaou N, Norekval TM, Spaulding C, Van Veldhuisen DJ (2015). 2015 ESC guidelines for the Management of Patients with ventricular arrhythmias and the prevention of sudden cardiac death: the task force for the management of patients with ventricular arrhythmias and the prevention of sudden cardiac death of the European Society of Cardiology (ESC)endorsed by: Association for European Paediatric and Congenital Cardiology (AEPC). Europace.

[27] Baskar S, Aziz PF (2015). Genotype-phenotype correlation in long QT syndrome. Glob Cardiol Sci Pract.

[28] Schwartz PJ, Ackerman MJ (2013). The long QT syndrome: a transatlantic clinical approach to diagnosis and therapy. Eur Heart J.

[29] Ackerman MJ, Priori SG, Willems S, Berul C, Brugada R, Calkins H, Camm AJ, Ellinor PT, Gollob M, Hamilton R, Hershberger RE, Judge DP, Le Marec H, McKenna WJ, Schulze-Bahr E, Semsarian C, Towbin JA, Watkins H, Wilde A, Wolpert C, Zipes DP (2011). HRS/EHRA expert consensus statement on the state of genetic testing for the channelopathies and cardiomyopathies this document was developed as a partnership between the Heart Rhythm Society (HRS) and the European heart rhythm association (EHRA). Heart Rhythm.

[30] Zareba W (2006). Genotype-specific ECG patterns in long QT syndrome. J Electrocardiol.

[31] Kaufman ES (2009). Mechanisms and clinical management of inherited channelopathies: long QT syndrome, Brugada syndrome, catecholaminergic polymorphic ventricular tachycardia, and short QT syndrome. Heart Rhythm.

[32] Bezzina CR, Lahrouchi N, Priori SG (2015). Genetics of sudden cardiac death. Circ Res.

[33] Kauferstein S, Kiehne N, Erkapic D, Schmidt J, Hamm CW, Bratzke H, Pitschner HF, Kuniss M, Neumann T (2011). A novel mutation in the cardiac ryanodine receptor gene (RyR2) in a patient with an unequivocal LQTS. Int J Cardiol.

[34] Altmann HM, Tester DJ, Will ML, Middha S, Evans JM, Eckloff BW, Ackerman MJ (2015). Homozygous/compound heterozygous Triadin mutations associated with Autosomal-recessive long-QT syndrome and pediatric sudden cardiac arrest: elucidation of the Triadin knockout syndrome. Circulation.

[35] Riuro H, Campuzano O, Arbelo E, Iglesias A, Batlle M, Perez-Villa F, Brugada J, Perez GJ, Scornik FS, Brugada R (2014). A missense mutation in the sodium channel beta1b subunit reveals SCN1B as a susceptibility gene underlying long QT syndrome. Heart Rhythm.

[36] Gaita F, Giustetto C, Bianchi F, Wolpert C, Schimpf R, Riccardi R, Grossi S, Richiardi E, Borggrefe M (2003). Short QT syndrome: a familial cause of sudden death. Circulation.

[37] Gussak I, Brugada P, Brugada J, Wright RS, Kopecky SL, Chaitman BR, Bjerregaard P (2000). Idiopathic short QT interval: a new clinical syndrome?. Cardiology.

[38] Priori SG, Wilde AA, Horie M, Cho Y, Behr ER, Berul C, Blom N, Brugada J, Chiang CE, Huikuri H, Kannankeril P, Krahn A, Leenhardt A, Moss A, Schwartz PJ, Shimizu W, Tomaselli G, Tracy C (2013). HRS/EHRA/APHRS expert consensus statement on the diagnosis and management of patients with inherited primary arrhythmia syndromes: document endorsed by HRS, EHRA, and APHRS in may 2013 and by ACCF, AHA, PACES, and AEPC in June 2013. Heart Rhythm.

[39] Mazzanti A, Kanthan A, Monteforte N, Memmi M, Bloise R, Novelli V, Miceli C, O’Rourke S, Borio G, Zienciuk-Krajka A, Curcio A, Surducan AE, Colombo M, Napolitano C, Priori SG (2014). Novel insight into the natural history of short QT syndrome. J Am Coll Cardiol.

[40] Antzelevitch C (2006). Brugada syndrome. Pacing Clin Electrophysiol.

[41] Juang JJ, Horie M (2016). Genetics of Brugada syndrome. J Arrhythm..

[42] Benito B, Sarkozy A, Mont L, Henkens S, Berruezo A, Tamborero D, Arzamendi D, Berne P, Brugada R, Brugada P, Brugada J (2008). Gender differences in clinical manifestations of Brugada syndrome. J Am Coll Cardiol.

[43] Nademanee K, Veerakul G, Nimmannit S, Chaowakul V, Bhuripanyo K, Likittanasombat K, Tunsanga K, Kuasirikul S, Malasit P, Tansupasawadikul S, Tatsanavivat P (1997). Arrhythmogenic marker for the sudden unexplained death syndrome in Thai men. Circulation.

[44] Priori SG, Napolitano C, Giordano U, Collisani G, Memmi M (2000). Brugada syndrome and sudden cardiac death in children. Lancet.

[45] Chen Q, Kirsch GE, Zhang D, Brugada R, Brugada J, Brugada P, Potenza D, Moya A, Borggrefe M, Breithardt G, Ortiz-Lopez R, Wang Z, Antzelevitch C, O’Brien RE, Schulze-Bahr E, Keating MT, Towbin JA, Wang Q (1998). Genetic basis and molecular mechanism for idiopathic ventricular fibrillation. Nature.

[46] Curcio A, Santarpia G, Indolfi C. The Brugada syndrome- from gene to therapy. Circ J. 2017;81(3):290–7. 10.1253/circj.CJ-16-0971 28070060

[47] Riuro H, Beltran-Alvarez P, Tarradas A, Selga E, Campuzano O, Verges M, Pagans S, Iglesias A, Brugada J, Brugada P, Vazquez FM, Perez GJ, Scornik FS, Brugada R (2013). A missense mutation in the sodium channel beta2 subunit reveals SCN2B as a new candidate gene for Brugada syndrome. Hum Mutat.

[48] Watanabe H, Koopmann TT, Le Scouarnec S, Yang T, Ingram CR, Schott JJ, Demolombe S, Probst V, Anselme F, Escande D, Wiesfeld AC, Pfeufer A, Kaab S, Wichmann HE, Hasdemir C, Aizawa Y, Wilde AA, Roden DM, Bezzina CR (2008). Sodium channel beta1 subunit mutations associated with Brugada syndrome and cardiac conduction disease in humans. J Clin Invest.

[49] Wu L, Yong SL, Fan C, Ni Y, Yoo S, Zhang T, Zhang X, Obejero-Paz CA, Rho HJ, Ke T, Szafranski P, Jones SW, Chen Q, Wang QK (2008). Identification of a new co-factor, MOG1, required for the full function of cardiac sodium channel Nav 1.5. J Biol Chem.

[50] Kattygnarath D, Maugenre S, Neyroud N, Balse E, Ichai C, Denjoy I, Dilanian G, Martins RP, Fressart V, Berthet M, Schott JJ, Leenhardt A, Probst V, Le Marec H, Hainque B, Coulombe A, Hatem SN, Guicheney P (2011). MOG1: a new susceptibility gene for Brugada syndrome. Circ Cardiovasc Genet.

[51] London B, Michalec M, Mehdi H, Zhu X, Kerchner L, Sanyal S, Viswanathan PC, Pfahnl AE, Shang LL, Madhusudanan M, Baty CJ, Lagana S, Aleong R, Gutmann R, Ackerman MJ, McNamara DM, Weiss R, Dudley SC (2007). Mutation in glycerol-3-phosphate dehydrogenase 1 like gene (GPD1-L) decreases cardiac Na+ current and causes inherited arrhythmias. Circulation.

[52] Cerrone M, Lin X, Zhang M, Agullo-Pascual E, Pfenniger A, Chkourko GH, Novelli V, Kim C, Tirasawadichai T, Judge DP, Rothenberg E, Chen HS, Napolitano C, Priori SG, Delmar M (2014). Missense mutations in plakophilin-2 cause sodium current deficit and associate with a Brugada syndrome phenotype. Circulation.

[53] Liu H, Chatel S, Simard C, Syam N, Salle L, Probst V, Morel J, Millat G, Lopez M, Abriel H, Schott JJ, Guinamard R, Bouvagnet P (2013). Molecular genetics and functional anomalies in a series of 248 Brugada cases with 11 mutations in the TRPM4 channel. PLoS One.

[54] Antzelevitch C, Pollevick GD, Cordeiro JM, Casis O, Sanguinetti MC, Aizawa Y, Guerchicoff A, Pfeiffer R, Oliva A, Wollnik B, Gelber P, Bonaros EP, Burashnikov E, Wu Y, Sargent JD, Schickel S, Oberheiden R, Bhatia A, Hsu LF, Haissaguerre M, Schimpf R, Borggrefe M, Wolpert C (2007). Loss-of-function mutations in the cardiac calcium channel underlie a new clinical entity characterized by ST-segment elevation, short QT intervals, and sudden cardiac death. Circulation.

[55] Antzelevitch C, Nof E (2008). Brugada syndrome: recent advances and controversies. Curr Cardiol Rep.

[56] Burashnikov E, Pfeiffer R, Barajas-Martinez H, Delpon E, Hu D, Desai M, Borggrefe M, Haissaguerre M, Kanter R, Pollevick GD, Guerchicoff A, Laino R, Marieb M, Nademanee K, Nam GB, Robles R, Schimpf R, Stapleton DD, Viskin S, Winters S, Wolpert C, Zimmern S, Veltmann C, Antzelevitch C (2010). Mutations in the cardiac L-type calcium channel associated with inherited J-wave syndromes and sudden cardiac death. Heart Rhythm.

[57] Sumitomo N (2016). Current topics in catecholaminergic polymorphic ventricular tachycardia. J Arrhythm.

[58] Priori SG, Napolitano C, Tiso N, Memmi M, Vignati G, Bloise R, Sorrentino V, Danieli GA (2001). Mutations in the cardiac ryanodine receptor gene (hRyR2) underlie catecholaminergic polymorphic ventricular tachycardia. Circulation.

[59] Devalla HD, Gelinas R, Aburawi EH, Beqqali A, Goyette P, Freund C, Chaix MA, Tadros R, Jiang H, Le Bechec A, Monshouwer-Kloots JJ, Zwetsloot T, Kosmidis G, Latour F, Alikashani A, Hoekstra M, Schlaepfer J, Mummery CL, Stevenson B, Kutalik Z, de Vries AA, Rivard L, Wilde AA, Talajic M, Verkerk AO, Al-Gazali L, Rioux JD, Bhuiyan ZA, Passier R (2016). TECRL, a new life-threatening inherited arrhythmia gene associated with overlapping clinical features of both LQTS and CPVT. EMBO Mol Med.

[60] Maron BJ, Maron MS, Semsarian C (2012). Genetics of hypertrophic cardiomyopathy after 20 years: clinical perspectives. J Am Coll Cardiol.

[61] Houston BA, Stevens GR (2014). Hypertrophic cardiomyopathy: a review. Clin Med Insights Cardiol.

[62] Sabater-Molina M, Saura D, Garcia-Molina SE, Gonzalez-Carrillo J, Polo L, Perez-Sanchez I, Olmo MD, Oliva-Sandoval MJ, Barriales-Villa R, Carbonell P, Pascual-Figal D, Gimeno JR (2017). A novel founder mutation in MYBPC3: phenotypic comparison with the most prevalent MYBPC3 mutation in Spain. Rev Esp Cardiol (Engl Ed).

[63] Osio A, Tan L, Chen SN, Lombardi R, Nagueh SF, Shete S, Roberts R, Willerson JT, Marian AJ (2007). Myozenin 2 is a novel gene for human hypertrophic cardiomyopathy. Circ Res.

[64] Chiu C, Bagnall RD, Ingles J, Yeates L, Kennerson M, Donald JA, Jormakka M, Lind JM, Semsarian C (2010). Mutations in alpha-actinin-2 cause hypertrophic cardiomyopathy: a genome-wide analysis. J Am Coll Cardiol.

[65] Weintraub RG, Semsarian C, Macdonald P. Dilated cardiomyopathy. Lancet. 2017;390(10092):400–14. 10.1016/S0140-6736(16)31713-5 28190577

[66] Michels VV, Moll PP, Miller FA, Tajik AJ, Chu JS, Driscoll DJ, Burnett JC, Rodeheffer RJ, Chesebro JH, Tazelaar HD (1992). The frequency of familial dilated cardiomyopathy in a series of patients with idiopathic dilated cardiomyopathy. N Engl J Med.

[67] Jefferies JL, Towbin JA (2010). Dilated cardiomyopathy. Lancet.

[68] Gerull B, Gramlich M, Atherton J, McNabb M, Trombitas K, Sasse-Klaassen S, Seidman JG, Seidman C, Granzier H, Labeit S, Frenneaux M, Thierfelder L (2002). Mutations of TTN, encoding the giant muscle filament titin, cause familial dilated cardiomyopathy. Nat Genet.

[69] Itoh-Satoh M, Hayashi T, Nishi H, Koga Y, Arimura T, Koyanagi T, Takahashi M, Hohda S, Ueda K, Nouchi T, Hiroe M, Marumo F, Imaizumi T, Yasunami M, Kimura A (2002). Titin mutations as the molecular basis for dilated cardiomyopathy. Biochem Biophys Res Commun.

[70] Herman DS, Lam L, Taylor MR, Wang L, Teekakirikul P, Christodoulou D, Conner L, DePalma SR, McDonough B, Sparks E, Teodorescu DL, Cirino AL, Banner NR, Pennell DJ, Graw S, Merlo M, Di Lenarda A, Sinagra G, Bos JM, Ackerman MJ, Mitchell RN, Murry CE, Lakdawala NK, Ho CY, Barton PJ, Cook SA, Mestroni L, Seidman JG, Seidman CE (2012). Truncations of titin causing dilated cardiomyopathy. N Engl J Med.

[71] Begay RL, Graw S, Sinagra G, Merlo M, Slavov D, Gowan K, Jones KL, Barbati G, Spezzacatene A, Brun F, Di Lenarda A, Smith JE, Granzier HL, Mestroni L, Taylor M. and Familial Cardiomyopathy R. Role of Titin Missense Variants in Dilated Cardiomyopathy. J Am Heart Assoc. 2015;4(11):1–9. 10.1161/JAHA.115.002645 PMC4845231 26567375

[72] Merlo M, Sinagra G, Carniel E, Slavov D, Zhu X, Barbati G, Spezzacatene A, Ramani F, Salcedo E, Di Lenarda A, Mestroni L, Taylor MR, Familial CR (2013). Poor prognosis of rare sarcomeric gene variants in patients with dilated cardiomyopathy. Clinical and translational science.

[73] Golbus JR, Puckelwartz MJ, Fahrenbach JP, Dellefave-Castillo LM, Wolfgeher D, McNally EM (2012). Population-based variation in cardiomyopathy genes. Circ Cardiovasc Genet.

[74] Schmitt JP, Kamisago M, Asahi M, Li GH, Ahmad F, Mende U, Kranias EG, MacLennan DH, Seidman JG, Seidman CE (2003). Dilated cardiomyopathy and heart failure caused by a mutation in phospholamban. Science.

[75] Fatkin D, MacRae C, Sasaki T, Wolff MR, Porcu M, Frenneaux M, Atherton J, Vidaillet HJ, Spudich S, De Girolami U, Seidman JG, Seidman C, Muntoni F, Muehle G, Johnson W, McDonough B (1999). Missense mutations in the rod domain of the lamin a/C gene as causes of dilated cardiomyopathy and conduction-system disease. N Engl J Med.

[76] Brodehl A, Ferrier RA, Hamilton SJ, Greenway SC, Brundler MA, Yu W, Gibson WT, McKinnon ML, McGillivray B, Alvarez N, Giuffre M, Schwartzentruber J, Gerull B (2016). Mutations in FLNC are associated with familial restrictive Cardiomyopathy. Hum Mutat.

[77] Elliott P, Andersson B, Arbustini E, Bilinska Z, Cecchi F, Charron P, Dubourg O, Kuhl U, Maisch B, McKenna WJ, Monserrat L, Pankuweit S, Rapezzi C, Seferovic P, Tavazzi L, Keren A (2008). Classification of the cardiomyopathies: a position statement from the European Society of Cardiology working group on myocardial and pericardial diseases. Eur Heart J.

[78] Rabbani B, Mahdieh N, Hosomichi K, Nakaoka H, Inoue I (2012). Next-generation sequencing: impact of exome sequencing in characterizing Mendelian disorders. J Hum Genet.

[79] Kostareva A, Kiselev A, Gudkova A, Frishman G, Ruepp A, Frishman D, Smolina N, Tarnovskaya S, Nilsson D, Zlotina A, Khodyuchenko T, Vershinina T, Pervunina T, Klyushina A, Kozlenok A, Sjoberg G, Golovljova I, Sejersen T, Shlyakhto E (2016). Genetic Spectrum of idiopathic restrictive Cardiomyopathy uncovered by next-generation sequencing. PLoS One.

[80] Gallego-Delgado M, Delgado JF, Brossa-Loidi V, Palomo J, Marzoa-Rivas R, Perez-Villa F, Salazar-Mendiguchia J, Ruiz-Cano MJ, Gonzalez-Lopez E, Padron-Barthe L, Bornstein B, Alonso-Pulpon L, Garcia-Pavia P (2016). Idiopathic restrictive Cardiomyopathy is primarily a genetic disease. J Am Coll Cardiol.

[81] Rani B, Kumar A, Bahl A, Sharma R, Prasad R, Khullar M (2017). Renin-angiotensin system gene polymorphisms as potential modifiers of hypertrophic and dilated cardiomyopathy phenotypes. Mol Cell Biochem.

[82] Perkins MJ, Van Driest SL, Ellsworth EG, Will ML, Gersh BJ, Ommen SR, Ackerman MJ (2005). Gene-specific modifying effects of pro-LVH polymorphisms involving the renin-angiotensin-aldosterone system among 389 unrelated patients with hypertrophic cardiomyopathy. Eur Heart J.

[83] Rai TS, Dhandapany PS, Ahluwalia TS, Bhardwaj M, Bahl A, Talwar KK, Nair K, Rathinavel A, Khullar M (2008). ACE I/D polymorphism in Indian patients with hypertrophic cardiomyopathy and dilated cardiomyopathy. Mol Cell Biochem.

[84] Osterop AP, Kofflard MJ, Sandkuijl LA, ten Cate FJ, Krams R, Schalekamp MA, Danser AH (1998). AT1 receptor a/C1166 polymorphism contributes to cardiac hypertrophy in subjects with hypertrophic cardiomyopathy. Hypertension.

[85] Azaouagh A, Churzidse S, Konorza T, Erbel R (2011). Arrhythmogenic right ventricular cardiomyopathy/dysplasia: a review and update. Clin Res Cardiol.

[86] Ohno S (2016). The genetic background of arrhythmogenic right ventricular cardiomyopathy. J Arrhythm..

[87] Ahmad F, Li D, Karibe A, Gonzalez O, Tapscott T, Hill R, Weilbaecher D, Blackie P, Furey M, Gardner M, Bachinski LL, Roberts R (1998). Localization of a gene responsible for arrhythmogenic right ventricular dysplasia to chromosome 3p23. Circulation.

[88] Corrado D, Thiene G (2006). Arrhythmogenic right ventricular cardiomyopathy/dysplasia: clinical impact of molecular genetic studies. Circulation.

[89] Protonotarios N, Tsatsopoulou A (2004). Naxos disease and Carvajal syndrome: cardiocutaneous disorders that highlight the pathogenesis and broaden the spectrum of arrhythmogenic right ventricular cardiomyopathy. Cardiovasc Pathol.

[90] McKoy G, Protonotarios N, Crosby A, Tsatsopoulou A, Anastasakis A, Coonar A, Norman M, Baboonian C, Jeffery S, McKenna WJ (2000). Identification of a deletion in plakoglobin in arrhythmogenic right ventricular cardiomyopathy with palmoplantar keratoderma and woolly hair (Naxos disease). Lancet.

[91] Pilichou K, Nava A, Basso C, Beffagna G, Bauce B, Lorenzon A, Frigo G, Vettori A, Valente M, Towbin J, Thiene G, Danieli GA, Rampazzo A (2006). Mutations in desmoglein-2 gene are associated with arrhythmogenic right ventricular cardiomyopathy. Circulation.

[92] Rampazzo A, Nava A, Malacrida S, Beffagna G, Bauce B, Rossi V, Zimbello R, Simionati B, Basso C, Thiene G, Towbin JA, Danieli GA (2002). Mutation in human desmoplakin domain binding to plakoglobin causes a dominant form of arrhythmogenic right ventricular cardiomyopathy. Am J Hum Genet.

[93] Syrris P, Ward D, Evans A, Asimaki A, Gandjbakhch E, Sen-Chowdhry S, McKenna WJ (2006). Arrhythmogenic right ventricular dysplasia/cardiomyopathy associated with mutations in the desmosomal gene desmocollin-2. Am J Hum Genet.

[94] Gerull B, Heuser A, Wichter T, Paul M, Basson CT, McDermott DA, Lerman BB, Markowitz SM, Ellinor PT, MacRae CA, Peters S, Grossmann KS, Drenckhahn J, Michely B, Sasse-Klaassen S, Birchmeier W, Dietz R, Breithardt G, Schulze-Bahr E, Thierfelder L (2004). Mutations in the desmosomal protein plakophilin-2 are common in arrhythmogenic right ventricular cardiomyopathy. Nat Genet.

[95] Groeneweg JA, Bhonsale A, James CA, te Riele AS, Dooijes D, Tichnell C, Murray B, Wiesfeld AC, Sawant AC, Kassamali B, Atsma DE, Volders PG, de Groot NM, de Boer K, Zimmerman SL, Kamel IR, van der Heijden JF, Russell SD, Jan Cramer M, Tedford RJ, Doevendans PA, van Veen TA, Tandri H, Wilde AA, Judge DP, van Tintelen JP, Hauer RN, Calkins H (2015). Clinical presentation, long-term follow-up, and outcomes of 1001 Arrhythmogenic right ventricular dysplasia/Cardiomyopathy patients and family members. Circ Cardiovasc Genet.

[96] Mazurek S, Kim GH (2017). Genetic and epigenetic regulation of arrhythmogenic cardiomyopathy. Biochim Biophys Acta.

[97] Merner ND, Hodgkinson KA, Haywood AF, Connors S, French VM, Drenckhahn JD, Kupprion C, Ramadanova K, Thierfelder L, McKenna W, Gallagher B, Morris-Larkin L, Bassett AS, Parfrey PS, Young TL (2008). Arrhythmogenic right ventricular cardiomyopathy type 5 is a fully penetrant, lethal arrhythmic disorder caused by a missense mutation in the TMEM43 gene. Am J Hum Genet.

[98] Chen SN, Gurha P, Lombardi R, Ruggiero A, Willerson JT, Marian AJ (2014). The hippo pathway is activated and is a causal mechanism for adipogenesis in arrhythmogenic cardiomyopathy. Circ Res.

[99] Kim GH (2013). MicroRNA regulation of cardiac conduction and arrhythmias. Translational research : the journal of laboratory and clinical medicine.

[100] van Opbergen CJ, Delmar M, van Veen TA (2017). Potential new mechanisms of pro-arrhythmia in arrhythmogenic cardiomyopathy: focus on calcium sensitive pathways. Neth Hear J.

[101] Hoedemaekers YM, Caliskan K, Michels M, Frohn-Mulder I, van der Smagt JJ, Phefferkorn JE, Wessels MW, ten Cate FJ, Sijbrands EJ, Dooijes D, Majoor-Krakauer DF (2010). The importance of genetic counseling, DNA diagnostics, and cardiologic family screening in left ventricular noncompaction cardiomyopathy. Circ Cardiovasc Genet.

[102] Maron BJ, Towbin JA, Thiene G, Antzelevitch C, Corrado D, Arnett D, Moss AJ, Seidman CE, Young JB (2006). Contemporary definitions and classification of the cardiomyopathies: an American Heart Association scientific statement from the council on clinical cardiology, heart failure and transplantation committee; quality of care and outcomes research and functional genomics and translational biology interdisciplinary working groups; and council on epidemiology and prevention. Circulation.

[103] Roston TM, Guo W, Krahn AD, Wang R, Van Petegem F, Sanatani S, Chen SR, Lehman A (2017). A novel RYR2 loss-of-function mutation (I4855M) is associated with left ventricular non-compaction and atypical catecholaminergic polymorphic ventricular tachycardia. J Electrocardiol.

[104] Klaassen S, Probst S, Oechslin E, Gerull B, Krings G, Schuler P, Greutmann M, Hurlimann D, Yegitbasi M, Pons L, Gramlich M, Drenckhahn JD, Heuser A, Berger F, Jenni R, Thierfelder L (2008). Mutations in sarcomere protein genes in left ventricular noncompaction. Circulation.

[105] Gazzerro E, Sotgia F, Bruno C, Lisanti MP, Minetti C (2010). Caveolinopathies: from the biology of caveolin-3 to human diseases. Eur J Hum Genet.

[106] Cagliani R, Bresolin N, Prelle A, Gallanti A, Fortunato F, Sironi M, Ciscato P, Fagiolari G, Bonato S, Galbiati S, Corti S, Lamperti C, Moggio M, Comi GP (2003). A CAV3 microdeletion differentially affects skeletal muscle and myocardium. Neurology.

[107] Catteruccia M, Sanna T, Santorelli FM, Tessa A, Di Giacopo R, Sauchelli D, Verbo A, Lo MM, Servidei S (2009). Rippling muscle disease and cardiomyopathy associated with a mutation in the CAV3 gene. Neuromuscul Disord.

[108] Traverso M, Gazzerro E, Assereto S, Sotgia F, Biancheri R, Stringara S, Giberti L, Pedemonte M, Wang X, Scapolan S, Pasquini E, Donati MA, Zara F, Lisanti MP, Bruno C, Minetti C (2008). Caveolin-3 T78M and T78K missense mutations lead to different phenotypes in vivo and in vitro. Lab Investig.

[109] Ricker K, Moxley RT, Rohkamm R (1989). Rippling muscle disease. Arch Neurol.

[110] Cheng J, Valdivia CR, Vaidyanathan R, Balijepalli RC, Ackerman MJ, Makielski JC (2013). Caveolin-3 suppresses late sodium current by inhibiting nNOS-dependent S-nitrosylation of SCN5A. J Mol Cell Cardiol.

[111] Barhanin J, Lesage F, Guillemare E, Fink M, Lazdunski M, Romey G (1996). K(V)LQT1 and lsK (minK) proteins associate to form the I(Ks) cardiac potassium current. Nature.

[112] Bellocq C, van Ginneken AC, Bezzina CR, Alders M, Escande D, Mannens MM, Baro I, Wilde AA (2004). Mutation in the KCNQ1 gene leading to the short QT-interval syndrome. Circulation.

[113] Wang Q, Curran ME, Splawski I, Burn TC, Millholland JM, VanRaay TJ, Shen J, Timothy KW, Vincent GM, de Jager T, Schwartz PJ, Toubin JA, Moss AJ, Atkinson DL, Landes GM, Connors TD, Keating MT (1996). Positional cloning of a novel potassium channel gene: KVLQT1 mutations cause cardiac arrhythmias. Nat Genet.

[114] Curran ME, Splawski I, Timothy KW, Vincent GM, Green ED, Keating MT (1995). A molecular basis for cardiac arrhythmia: HERG mutations cause long QT syndrome. Cell.

[115] Wang Q, Shen J, Splawski I, Atkinson D, Li Z, Robinson JL, Moss AJ, Towbin JA, Keating MT (1995). SCN5A mutations associated with an inherited cardiac arrhythmia, long QT syndrome. Cell.

[116] Mohler PJ, Schott JJ, Gramolini AO, Dilly KW, Guatimosim S, duBell WH, Song LS, Haurogne K, Kyndt F, Ali ME, Rogers TB, Lederer WJ, Escande D, Le Marec H, Bennett V (2003). Ankyrin-B mutation causes type 4 long-QT cardiac arrhythmia and sudden cardiac death. Nature.

[117] Splawski I, Tristani-Firouzi M, Lehmann MH, Sanguinetti MC, Keating MT (1997). Mutations in the hminK gene cause long QT syndrome and suppress IKs function. Nat Genet.

[118] Kaczmarek LK, Blumenthal EM (1997). Properties and regulation of the minK potassium channel protein. Physiol Rev.

[119] Abbott GW, Sesti F, Splawski I, Buck ME, Lehmann MH, Timothy KW, Keating MT, Goldstein SA (1999). MiRP1 forms IKr potassium channels with HERG and is associated with cardiac arrhythmia. Cell.

[120] Fodstad H, Swan H, Auberson M, Gautschi I, Loffing J, Schild L, Kontula K (2004). Loss-of-function mutations of the K(+) channel gene KCNJ2 constitute a rare cause of long QT syndrome. J Mol Cell Cardiol.

[121] Boczek NJ, Best JM, Tester DJ, Giudicessi JR, Middha S, Evans JM, Kamp TJ, Ackerman MJ (2013). Exome sequencing and systems biology converge to identify novel mutations in the L-type calcium channel, CACNA1C, linked to autosomal dominant long QT syndrome. Circ Cardiovasc Genet.

[122] Medeiros-Domingo A, Kaku T, Tester DJ, Iturralde-Torres P, Itty A, Ye B, Valdivia C, Ueda K, Canizales-Quinteros S, Tusie-Luna MT, Makielski JC, Ackerman MJ (2007). SCN4B-encoded sodium channel beta4 subunit in congenital long-QT syndrome. Circulation.

[123] Chen L, Marquardt ML, Tester DJ, Sampson KJ, Ackerman MJ, Kass RS (2007). Mutation of an A-kinase-anchoring protein causes long-QT syndrome. Proc Natl Acad Sci U S A.

[124] Ueda K, Valdivia C, Medeiros-Domingo A, Tester DJ, Vatta M, Farrugia G, Ackerman MJ, Makielski JC (2008). Syntrophin mutation associated with long QT syndrome through activation of the nNOS-SCN5A macromolecular complex. Proc Natl Acad Sci U S A.

[125] Wang F, Liu J, Hong L, Liang B, Graff C, Yang Y, Christiansen M, Olesen SP, Zhang L, Kanters JK (2013). The phenotype characteristics of type 13 long QT syndrome with mutation in KCNJ5 (Kir3.4-G387R). Heart Rhythm.

[126] Crotti L, Johnson CN, Graf E, De Ferrari GM, Cuneo BF, Ovadia M, Papagiannis J, Feldkamp MD, Rathi SG, Kunic JD, Pedrazzini M, Wieland T, Lichtner P, Beckmann BM, Clark T, Shaffer C, Benson DW, Kaab S, Meitinger T, Strom TM, Chazin WJ, Schwartz PJ, George AL (2013). Calmodulin mutations associated with recurrent cardiac arrest in infants. Circulation.

[127] Makita N, Yagihara N, Crotti L, Johnson CN, Beckmann BM, Roh MS, Shigemizu D, Lichtner P, Ishikawa T, Aiba T, Homfray T, Behr ER, Klug D, Denjoy I, Mastantuono E, Theisen D, Tsunoda T, Satake W, Toda T, Nakagawa H, Tsuji Y, Tsuchiya T, Yamamoto H, Miyamoto Y, Endo N, Kimura A, Ozaki K, Motomura H, Suda K, Tanaka T, Schwartz PJ, Meitinger T, Kaab S, Guicheney P, Shimizu W, Bhuiyan ZA, Watanabe H, Chazin WJ, George AL Jr, et al. Novel calmodulin mutations associated with congenital arrhythmia susceptibility. Circ Cardiovasc Genet. 2014;7(4):466–74. 10.1161/CIRCGENETICS.113.000459 PMC4140998 24917665

[128] Reed GJ, Boczek NJ, Etheridge SP, Ackerman MJ (2015). CALM3 mutation associated with long QT syndrome. Heart Rhythm.

[129] Brugada R, Hong K, Dumaine R, Cordeiro J, Gaita F, Borggrefe M, Menendez TM, Brugada J, Pollevick GD, Wolpert C, Burashnikov E, Matsuo K, Wu YS, Guerchicoff A, Bianchi F, Giustetto C, Schimpf R, Brugada P, Antzelevitch C (2004). Sudden death associated with short-QT syndrome linked to mutations in HERG. Circulation.

[130] Priori SG, Pandit SV, Rivolta I, Berenfeld O, Ronchetti E, Dhamoon A, Napolitano C, Anumonwo J, di Barletta MR, Gudapakkam S, Bosi G, Stramba-Badiale M, Jalife J (2005). A novel form of short QT syndrome (SQT3) is caused by a mutation in the KCNJ2 gene. Circ Res.

[131] Templin C, Ghadri JR, Rougier JS, Baumer A, Kaplan V, Albesa M, Sticht H, Rauch A, Puleo C, Hu D, Barajas-Martinez H, Antzelevitch C, Luscher TF, Abriel H, Duru F (2011). Identification of a novel loss-of-function calcium channel gene mutation in short QT syndrome (SQTS6). Eur Heart J.

[132] Wehrens XH (2007). The molecular basis of catecholaminergic polymorphic ventricular tachycardia: what are the different hypotheses regarding mechanisms?. Heart Rhythm.

[133] Bhuiyan ZA, Hamdan MA, Shamsi ET, Postma AV, Mannens MM, Wilde AA, Al-Gazali L (2007). A novel early onset lethal form of catecholaminergic polymorphic ventricular tachycardia maps to chromosome 7p14-p22. J Cardiovasc Electrophysiol.

[134] Nyegaard M, Overgaard MT, Sondergaard MT, Vranas M, Behr ER, Hildebrandt LL, Lund J, Hedley PL, Camm AJ, Wettrell G, Fosdal I, Christiansen M, Borglum AD (2012). Mutations in calmodulin cause ventricular tachycardia and sudden cardiac death. Am J Hum Genet.

[135] Gomez-Hurtado N, Boczek NJ, Kryshtal DO, Johnson CN, Sun J, Nitu FR, Cornea RL, Chazin WJ, Calvert ML, Tester DJ, Ackerman MJ, Knollmann BC Novel CPVT-Associated Calmodulin Mutation in CALM3 (CALM3-A103V) Activates Arrhythmogenic Ca Waves and Sparks. Circ Arrhythm Electrophysiol. 2016;9(8):1–22. 10.1161/CIRCEP.116.004161 PMC4988333 27516456

[136] Beffagna G, Occhi G, Nava A, Vitiello L, Ditadi A, Basso C, Bauce B, Carraro G, Thiene G, Towbin JA, Danieli GA, Rampazzo A (2005). Regulatory mutations in transforming growth factor-beta3 gene cause arrhythmogenic right ventricular cardiomyopathy type 1. Cardiovasc Res.

[137] Leask A, Abraham DJ (2004). TGF-beta signaling and the fibrotic response. FASEB J.

[138] Rampazzo A, Nava A, Erne P, Eberhard M, Vian E, Slomp P, Tiso N, Thiene G, Danieli GA (1995). A new locus for arrhythmogenic right ventricular cardiomyopathy (ARVD2) maps to chromosome 1q42-q43. Hum Mol Genet.

[139] Tiso N, Stephan DA, Nava A, Bagattin A, Devaney JM, Stanchi F, Larderet G, Brahmbhatt B, Brown K, Bauce B, Muriago M, Basso C, Thiene G, Danieli GA, Rampazzo A (2001). Identification of mutations in the cardiac ryanodine receptor gene in families affected with arrhythmogenic right ventricular cardiomyopathy type 2 (ARVD2). Hum Mol Genet.

[140] Severini GM, Krajinovic M, Pinamonti B, Sinagra G, Fioretti P, Brunazzi MC, Falaschi A, Camerini F, Giacca M, Mestroni L (1996). A new locus for arrhythmogenic right ventricular dysplasia on the long arm of chromosome 14. Genomics.

[141] Taylor M, Graw S, Sinagra G, Barnes C, Slavov D, Brun F, Pinamonti B, Salcedo EE, Sauer W, Pyxaras S, Anderson B, Simon B, Bogomolovas J, Labeit S, Granzier H, Mestroni L (2011). Genetic variation in titin in arrhythmogenic right ventricular cardiomyopathy-overlap syndromes. Circulation.

[142] Li D, Ahmad F, Gardner MJ, Weilbaecher D, Hill R, Karibe A, Gonzalez O, Tapscott T, Sharratt GP, Bachinski LL, Roberts R (2000). The locus of a novel gene responsible for arrhythmogenic right-ventricular dysplasia characterized by early onset and high penetrance maps to chromosome 10p12-p14. Am J Hum Genet.

[143] Klauke B, Kossmann S, Gaertner A, Brand K, Stork I, Brodehl A, Dieding M, Walhorn V, Anselmetti D, Gerdes D, Bohms B, Schulz U, Zu KE, Vorgerd M, Gummert J, Milting H (2010). De novo desmin-mutation N116S is associated with arrhythmogenic right ventricular cardiomyopathy. Hum Mol Genet.

[144] Syrris P, Ward D, Asimaki A, Sen-Chowdhry S, Ebrahim HY, Evans A, Hitomi N, Norman M, Pantazis A, Shaw AL, Elliott PM, McKenna WJ (2006). Clinical expression of plakophilin-2 mutations in familial arrhythmogenic right ventricular cardiomyopathy. Circulation.

[145] van der Zwaag PA, van Rijsingen IA, Asimaki A, Jongbloed JD, van Veldhuisen DJ, Wiesfeld AC, Cox MG, van Lochem LT, de Boer RA, Hofstra RM, Christiaans I, van Spaendonck-Zwarts KY, Lekanne dit Deprez RH, Judge DP, Calkins H, Suurmeijer AJ, Hauer RN, Saffitz JE, Wilde AA, Van den Berg MP, van Tintelen JP (2012). Phospholamban R14del mutation in patients diagnosed with dilated cardiomyopathy or arrhythmogenic right ventricular cardiomyopathy: evidence supporting the concept of arrhythmogenic cardiomyopathy. Eur J Heart Fail.

[146] Quarta G, Syrris P, Ashworth M, Jenkins S, Zuborne AK, Morgan J, Muir A, Pantazis A, McKenna WJ, Elliott PM (2012). Mutations in the Lamin a/C gene mimic arrhythmogenic right ventricular cardiomyopathy. Eur Heart J.

[147] Erkapic D, Neumann T, Schmitt J, Sperzel J, Berkowitsch A, Kuniss M, Hamm CW, Pitschner HF (2008). Electrical storm in a patient with arrhythmogenic right ventricular cardiomyopathy and SCN5A mutation. Europace.

[148] van Hengel J, Calore M, Bauce B, Dazzo E, Mazzotti E, De Bortoli M, Lorenzon A, Li Mura IE, Beffagna G, Rigato I, Vleeschouwers M, Tyberghein K, Hulpiau P, van Hamme E, Zaglia T, Corrado D, Basso C, Thiene G, Daliento L, Nava A, van Roy F, Rampazzo A (2013). Mutations in the area composita protein alphaT-catenin are associated with arrhythmogenic right ventricular cardiomyopathy. Eur Heart J.

